# Factors associated with probable sarcopenic dysphagia and development of a clinical classification model in older ICU patients: a cross-sectional study

**DOI:** 10.3389/fmed.2026.1910425

**Published:** 2026-10-07

**Authors:** Xiaoping Shao, Zixin Wang, Jie Li, Xianshuai Wu, Xiaoyan Zhu, Qunfeng Lu

**Affiliations:** Department of Nursing, Shanghai Sixth People's Hospital Affiliated to Shanghai Jiao Tong University School of Medicine, Shanghai, China

**Keywords:** intensive care unit, nomogram, prediction model, sarcopenic dysphagia, ultrasonography

## Abstract

**Objective:**

To analyze factors associated with probable sarcopenic dysphagia in older ICU patients, develop a clinical classification model, and provide evidence for early bedside identification and targeted intervention.

**Methods:**

A cross-sectional study was conducted with 160 older patients admitted to a tertiary ICU from January to December 2025 via convenience sampling. Patients were grouped using the Ishii sarcopenia screening score and the Modified Water Swallowing Test (MWST). Data collected included general patient characteristics, ultrasound measurements of swallowing-related muscles, gastric motility ultrasound parameters, functional status indicators, and nutritional risk screening results. Univariate analysis and binary logistic regression were applied to identify factors associated with the outcome. A classification model was constructed and visualized as a nomogram using R software.

**Results:**

The prevalence of probable sarcopenic dysphagia among older ICU patients was 65.62%. Multivariable logistic regression showed that NRS 2002 score ≥ 3, severe genioglossus muscle thinning, presence of comorbidities, abnormal vocalization, and voluntary cough ability score ≤ 2 were independently associated with higher odds of probable sarcopenic dysphagia. The apparent area under the receiver operating characteristic curve (AUC) was 0.837 (95% CI: 0.775–0.900), and the bias-corrected AUC after bootstrap internal validation was 0.812.

**Conclusion:**

Probable sarcopenic dysphagia is highly prevalent in older ICU patients and is associated with nutritional status, swallowing muscle morphology, comorbidity burden, vocal function, and airway protective capacity. The classification model demonstrates good discrimination, calibration, and clinical utility, and may serve as a bedside tool for risk stratification. Multicenter external validation is required before clinical implementation.

## 1 Introduction

Sarcopenic dysphagia (SD) is swallowing difficulty caused by involvement of swallowing-related muscles in generalized sarcopenia, leading to decreased swallowing muscle mass and function (1, 2). SD is associated with adverse outcomes, including reduced nutritional intake, increased risk of aspiration pneumonia, decreased overall function, and increased mortality risk (3, 4). Sarcopenic dysphagia is a common yet underestimated syndrome in the elderly, especially among the very old, hospitalized, or institutionalized elderly. Research (5) has found that the incidence of sarcopenic dysphagia in institutionalized elderly is 45%. The ICU environment, through factors such as mechanical ventilation, sedation, prolonged immobilization, and high metabolic stress, disrupts the structure and neural coordination of swallowing-related muscles, significantly increasing the risk of sarcopenic dysphagia in elderly patients (6). Currently, research on sarcopenic dysphagia in China is still in its early stages, lacking epidemiological data (7).

Based on a review of SD-related literature (3, 4, 8, 9), this study investigated the factors influencing SD in elderly ICU patients and developed a prediction model to inform the development of preventive and intervention measures for high-risk populations.

## 2 Subjects and methods

### 2.1 Study design and participants

A cross-sectional design was employed. Older adult patients admitted to the ICU of a tertiary first-class hospital from January 2025 to December 2025 were recruited via convenience sampling.

#### 2.1.1 Inclusion criteria

① Age ≥60 years; ② ICU length of stay ≥48 hours; ③ Alert and cooperative with assessments, or hemodynamically stable and able to complete assessment with medical staff assistance; ④ Complete clinical data, including swallowing function, nutritional status, and muscle strength assessment data; ⑤ No history of esophageal or head/neck surgery or organic neurological disease.

#### 2.1.2 Exclusion criteria

① Severe disturbance of consciousness, delirium, or dementia preventing cooperation; ② History of oral cancer, laryngeal cancer, or head/neck radiotherapy directly affecting swallowing anatomy. ③ Acute stroke, acute brain injury, or critical illness polyneuropathy/myopathy confirmed by imaging or clinical diagnosis; ④ Pre-existing dysphagia diagnosed before ICU admission.

All patients were systematically screened for the above exclusion conditions through admission medical records, cranial imaging reports, and neurological examination performed by attending physicians, to rule out major non-sarcopenic causes of dysphagia of neurological or structural origin.

#### 2.1.3 Sample size calculation

According to sample size calculations for logistic regression (10), a minimum of 5–10 outcome events per independent variable is required. Conservatively, using EPV = 10 and an expected positive rate *P* = 0.50, the required sample size is: *n* = (m * EPV)/P, where m is the number of independent variables in the final model, EPV is the minimum number of events per variable required, and P is the expected positive rate. Substituting m = 8, EPV = 10, and *P* = 0.50 yields: *n* = (8 * 10)/0.50 = 160 cases.

The 8 candidate variables were prespecified based on literature review and expert consultation before data analysis. In the final model, 10 candidate variables with dummy coding for 3-category predictors resulted in 12 regression parameters, with an events-per-variable (EPV) ratio of approximately 8.75.

### 2.2 Ethical considerations

This study was approved by the hospital ethics committee: 2025-KY-425 (K). All participants provided informed consent and voluntarily participated.

### 2.3 Instruments

#### 2.3.1 General information questionnaire

Demographic data (age, sex, body mass index [BMI]) and clinical characteristics (primary diagnosis, comorbidities, mechanical ventilation status, ICU length of stay, serum albumin level) were collected from electronic medical records. Presence of comorbidities was defined as having ≥ 2 chronic underlying diseases, including diabetes mellitus, chronic obstructive pulmonary disease, chronic heart failure, chronic kidney disease, and hypertension.

#### 2.3.2 Swallowing function assessment

The Modified Water Swallowing Test (MWST) was used for screening. Patients were seated or semi-recumbent and asked to drink 30 mL of warm water. The time taken, presence of coughing, and whether swallowing required multiple attempts were observed and recorded. Grading criteria (11):

1. Grade I: Successful swallow in one attempt
2. Grade II: Swallow in two or more attempts without coughing
3. Grade III: Swallow in one attempt with coughing
4. Grade IV: Swallow in two or more attempts with coughing
5. Grade V: Frequent coughing, unable to swallow completely

Grade I was defined as normal swallowing function; Grades II–V indicated dysphagia.

All MWST assessments were performed by two trained ICU nurses within 24–48 hours of ICU admission after vital signs stabilized. Assessors were blinded to Ishii score and ultrasound findings. Testing was stopped immediately if oxygen desaturation, severe choking, or hemodynamic instability occurred.

#### 2.3.3 Sarcopenia screening questionnaire

The Ishii score (12) was used. This score combines age, grip strength, and calf circumference. Male score = 0.62 × (age-64) – 3.09 × (grip strength-50) – 4.64 × (calf circumference-42); Female score = 0.80 × (age-64) – 5.09 × (grip strength-34) – 3.28 × (calf circumference-42). A score above the cutoff (male >105, female >120) indicated sarcopenia. Grip strength was measured with an electronic dynamometer, and the maximum value from the dominant hand was recorded. Calf circumference was measured at the thickest part of the gastrocnemius muscle.

#### 2.3.4 Diagnostic criteria and study grouping

Per the 19th Annual Meeting consensus of the Japanese Society of Dysphagia Rehabilitation, definitive diagnostic criteria for sarcopenic dysphagia include: (1) presence of dysphagia; (2) presence of generalized sarcopenia; (3) imaging evidence of swallowing muscle mass loss; (4) exclusion of other causes of dysphagia; and (5) confirmation that sarcopenia is the primary cause (2).

Given the practical constraints of bedside ICU assessment, this study adopted an operational definition of probable sarcopenic dysphagia for screening purposes. Patients with both a positive Ishii sarcopenia screen and MWST grade ≥ II were assigned to the probable SD group (case group). Patients not meeting both criteria were assigned to the control group.

Notably, genioglossus muscle ultrasound was not used for outcome classification and was analyzed only as an independent predictor to avoid incorporation bias. This operational definition does not represent a definitive etiologic diagnosis, and outcome misclassification may exist.

#### 2.3.5 Ultrasound assessment of swallowing-related muscles

A portable ultrasound diagnostic device (linear probe, 5–12 MHz) was used. Patients were supine with the head in a neutral position. The maximum thickness of the following muscles was measured at rest, with each measurement repeated three times and averaged:

1. Genioglossus muscle thickness: Measured in the midsagittal plane of the floor of the mouth, from the inferior mandibular border to the dorsal genioglossus surface.
2. Mylohyoid muscle thickness: Measured in the parasagittal plane perpendicular to muscle fiber direction.

Thickness categories were defined based on published reference values for Chinese older adults: ≥ 8.0 mm = normal, 6.0–7.9 mm = mild thinning, < 6.0 mm = severe thinning. All scans were performed by two trained sonographers. Formal inter-rater and intra-rater reliability testing was not performed in this study, which may introduce measurement uncertainty.

#### 2.3.6 Ultrasound assessment of gastric antral motility

Abdominal ultrasound was used to assess gastric motility. Patients were placed in the right lateral decubitus position. Gastric antral contractions were observed and recorded over 3 minutes in a transverse section of the gastric antrum. Measured parameters included: (1) Gastric antral contraction frequency (times per minute); (2) Gastric antral contraction amplitude (%); (3) Gastric motility index.

Gastric motility index was graded as: normal (≥ 12 contractions/3 min with amplitude > 30%), mild reduction (8–11 contractions/3 min or amplitude 20–30%), and severe failure (< 8 contractions/3 min or amplitude < 20%), based on standard gastrointestinal ultrasound criteria.

#### 2.3.7 Functional status assessment

1. Voluntary cough ability was assessed using the semi-quantitative cough strength score (0–5 points) (13). Patients were asked to cough as hard as possible. Scores of 0–2 indicated weak cough strength and a high aspiration risk; scores of 3–5 indicated strong cough strength and adequate airway protection.
2. Vocal function was assessed using the GRBAS voice scale (14), which evaluates five dimensions: Grade (overall hoarseness), Roughness, Breathiness, Asthenia, and Strain. Each dimension is graded from 0 (normal) to 3 (severely abnormal). Higher total scores indicate more severe voice impairment, a total score ≥ 3 was defined as abnormal vocalization.
3. Lip and oral motor function was assessed using a lip and oral motor function assessment scale that evaluates seven items, including lip and tongue morphology, drooling, muscle strength, and mobility. The total score ranges from 0 to 28, with lower scores indicating more severe dysfunction.

#### 2.3.8 Nutritional risk screening 2002 (NRS 2002) scale

The NRS 2002, developed by the European Society for Clinical Nutrition and Metabolism (ESPEN) in 2002, is an evidence-based nutrition screening tool. The scale includes three dimensions: nutritional status impairment score (0–3 points), disease severity score (0–3 points), and age score (0–1 point), used to assess nutritional risk in adult inpatients. The total score ranges from 0 to 7. A total score ≥3 indicates nutritional risk; a total score <3 indicates no current nutritional risk. The Cronbach's *α* coefficient for the NRS 2002 was 0.779, and the Kappa value ranged from 0.551 to 0.813, indicating good reliability and validity (15).

### 2.4 Data collection and quality control

Trained operators used standardized language to guide patients in one-on-one sessions. When patients had difficulty understanding items due to educational level or physiological limitations, operators explained the content in detail without leading suggestions and assisted with completion. Questionnaires were collected and verified on the spot. Any missing items were completed immediately. Questionnaires with contradictory or logically erroneous answers were excluded. Data were entered and verified independently by two people.

### 2.5 Statistical methods

SPSS 27.0 and R 4.4.1 were used for data analysis. Categorical variables are presented as frequencies and percentages; between-group comparisons were performed using *χ*^2^ tests or Fisher's exact test as appropriate. Non-normally distributed continuous variables are presented as median (interquartile range) and compared using Mann–Whitney U tests.

Binary logistic regression was used for variable screening and model construction. Candidate predictors with *P* < 0.05 in univariate analysis were entered into the multivariable model using the enter method. Multicollinearity was assessed via variance inflation factor (VIF); all VIF values were < 2.5, indicating no significant multicollinearity. Primary analysis used categorized predictors for clinical utility; a sensitivity analysis using continuous predictors (genioglossus thickness in mm, NRS 2002 as integer score) was also performed to verify robustness.

Model discrimination was evaluated using the area under the ROC curve (AUC). Internal validation was performed via bootstrap resampling with 1,000 repetitions; the full model-building process was repeated in each sample to estimate optimism. Both apparent (original sample) and bias-corrected performance metrics are reported.

Model calibration was assessed using calibration curves, mean absolute error, calibration intercept, and calibration slope. Decision curve analysis (DCA) was used to evaluate clinical net benefit. A nomogram was constructed for bedside application. A two-tailed *P* < 0.05 was considered statistically significant. Predictor selection was partially data-driven based on univariate screening; thus the model is exploratory in nature.

## 3 Results

### 3.1 General information

A total of 198 patients were assessed for eligibility. Of these, 38 were excluded: 12 for severe consciousness disturbance, 9 for history of head and neck surgery/radiotherapy, 8 for acute stroke or acute neurological disease, 5 for pre-existing dysphagia, and 4 for incomplete clinical data. Finally, 160 patients were included in the analysis, comprising 93 males (58.1%) and 67 females (41.9%), with a mean age of 75.9 ± 4.9 years.

Per the operational definition, 105 patients met criteria for probable sarcopenic dysphagia (prevalence 65.6%), and 55 were in the control group. Among all participants, 37 (23.1%) had negative results on both screenings, 18 (11.3%) tested positive only on sarcopenia screening, 20 (12.5%) tested positive only on dysphagia screening, and 105 (65.6%) tested positive on both. No missing data were observed for model variables among the 160 included patients.

### 3.2 Univariate analysis of factors associated with probable sarcopenic dysphagia

Univariate analysis of variables showing statistical differences indicated that mechanical ventilation, tracheostomy, NRS 2002 score, genioglossus muscle thickness, gastric motility index, voluntary cough ability, presence of enteral/parenteral nutrition, presence of indwelling gastric tube, presence of comorbidities, and vocalization status were all associated with the occurrence of sarcopenic dysphagia (*P* < 0.05) (Table 1).

**Table 1:** Comparison of categorical data between the probable sarcopenic dysphagia group and control group [*n* (%)].

Item	Sarcopenic dysphagia	Control	*χ*^2^/z	*p*
	(*n* = 105)	(*n* = 55)		
Gender			1.850	0.174
Male	57 (54.3%)	36 (65.5%)		
Female	48 (45.7%)	19 (34.5%)		
Age			0.049	0.824
60∼79	42 (40.0%)	23 (41.8%)		
≥80	63 (60.0%)	32 (58.2%)		
BMI (kg/m^2^)			1.300	0.522
≤18	33 (31.4%)	18 (32.8%)		
18–24.9	49 (46.6%)	29 (52.7%)		
≥25	23 (21.9%)	8 (14.5%)		
Mechanical ventilation			5.252	0.022*
Yes	44 (41.9%)	13 (23.6%)		
No	61 (58.1%)	42 (76.4%)		
Tracheotomy			7.315	0.007**
Yes	34 (32.4%)	7 (12.7%)		
No	71 (67.6%)	48 (87.3%)		
Nasogastric tube placement			5.623	0.018*
Yes	31 (29.5%)	7 (12.7%)		
No	74 (70.5%)	48 (87.3%)		
Presence of comorbidities			12.799	0.001**
Yes	71 (67.6%)	21 (38.2%)		
No	34 (32.4%)	34 (61.8%)		
NRS 2002			5.676	0.017*
≥3	59 (56.2%)	20 (36.4%)		
≤3	46 (43.8%)	35 (63.6%)		
Diet type			3.314	0.191
Liquid	15 (14.3%)	14 (25.5%)		
Low fat and salt	55 (52.4%)	23 (41.8%)		
Regular	35 (33.3%)	18 (32.7%)		
Enteral/parenteral nutrition			6.191	0.013*
Yes	58 (55.2%)	19 (34.5%)		
No	47 (44.8%)	36 (65.5%)		
Number of missing teeth			2.811	0.245
≤5	32 (30.5%)	22 (40.0%)		
5–10	42 (40.0%)	23 (41.8%)		
≥11	31 (29.5%)	10 (18.2%)		
Voluntary cough ability			12.964	0.003**
≤2	62 (59.0%)	16 (29.1%)		
>2	43 (41.0%)	39 (70.9%)		
Vocalization status			9.578	0.002**
Normal	38 (36.2%)	34 (61.8%)		
Abnormal	67 (63.8%)	21 (38.2%)		
Lip/oral motor function			1.08	0.299
Normal	77 (73.3%)	36 (65.5%)		
No function	28 (26.7%)	19 (34.5%)		
Mylohyoid muscle thickness			2.417	0.299
Normal	45 (42.9%)	17 (31.0%)		
Mild thinning	33 (31.4%)	19 (34.5%)		
Severe thinning	27 (25.7%)	19 (34.5%)		
Genioglossus muscle thickness			12.205	0.002**
Normal	49 (46.7%)	11 (20.0%)		
Mild thinning	22 (21.0%)	22 (40.0%)		
Severe thinning	34 (32.3%)	22 (40.0%)		
Gastric antral contraction frequency			0.163	0.922
Normal	28 (26.7%)	14 (25.5%)		
Motility disorder	50 (47.6%)	28 (50.9%)		
Motility failure	27 (25.7%)	13 (23.6%)		
Gastric reduction index			0.309	0.857
Normal	25 (23.8%)	11 (20.0%)		
Mild reduction	57 (54.3%)	31 (56.4%)		
Severe/no contraction	23 (21.9%)	13 (23.6%)		
Gastric motility index			7.212	0.027*
Normal	13 (23.6%)	43 (41.0%)		
Decreased gastric motility	16 (29.1%)	33 (31.4%)		
Gastric motility failure	26 (47.3%)	29 (27.6%)		
Serum albumin (g/L)			0.11	0.947
30∼55	19 (18.1%)	9 (16.4%)		
20∼29	68 (64.8%)	37 (67.3%)		
≤20	18 (17.1%)	9 (16.3%)		
ICU length of stay (day)	11 (7∼14)	10 (7∼12)	−0.745	0.456

ICU, intensive care unit.

* *p* ≤ 0.05.

** *p* ≤ 0.01.

### 3.3 Multivariable logistic regression analysis of associated factors

Variables with *P* < 0.05 in univariate analysis were entered into the binary logistic regression model: mechanical ventilation, tracheostomy, NRS 2002 score, genioglossus muscle thickness, gastric motility index, voluntary cough ability, enteral/parenteral nutrition, nasogastric tube placement, comorbidities, and vocalization status. Variable coding is shown in Table 2.

**Table 2:** Assignment of independent variables.

Variable	Value
Probable sarcopenic dysphagia (outcome)	0 = No, 1 = Yes
Mechanical ventilation	0 = No, 1 = Yes
Tracheotomy	0 = No, 1 = Yes
NRS 2002	0=≤3, 1=≥3
Genioglossus muscle thickness	1= Severe Thinning, 2= Mild thinning, 3 = Normal
Gastric motility index	1 = Severe/no contraction 2 = Mild reduction, 3 = Normal
Voluntary cough ability	1=≤2 2=＞2
Enteral/parental nutrition	0 = No, 1 = Yes
Nasogastric tube placement	0 = No, 1 = Yes
Presence of comorbidities	0 = No, 1 = Yes
Vocalization status	0 = Normal, 1 = Abnormal

**Table:** 

Item	β	SE	Wald*χ*^2^	*P*	OR	95% CI
Constant	−4.551	0.959	22.544	<0.001	0.011	–
Presence of comorbidities	1.385	0.483	8.238	0.004	3.995	1.552∼10.287
Voluntary cough ability ≤2	1.988	0.531	14.019	<0.001	7.298	2.578∼20.658
Abnormal vocalization	1.647	0.496	11.037	0.001	5.192	1.965∼13.720
Severe genioglossus muscle thinness	1.971	0.618	10.163	0.001	7.177	2.137∼24.110
NRS 2002 ≤ 3	1.332	0.487	7.469	0.006	3.789	1.458∼9.849

Multivariable analysis showed that NRS 2002 score ≥ 3, severe genioglossus muscle thinning, presence of comorbidities, abnormal vocalization, and voluntary cough ability score ≤ 2 were independently associated with higher odds of probable sarcopenic dysphagia (all *P* < 0.05) (Table 3). Sensitivity analysis using continuous predictors yielded consistent directionality and significance of associations.

**Table 3:** Binary logistic regression analysis of influencing factors for sarcopenic dysphagia in elderly ICU patients (*n* = 160).

Variable	*β*	SE	Wald*χ*^2^	*P*	OR	95% CI
Intercept	−4.551	0.959	22.544	<0.001	0.011	–
Presence of comorbidities (yes vs. no)	1.385	0.483	8.238	0.004	3.995	1.552∼10.287
Voluntary cough ability ≤2 (vs. > 2)	1.988	0.531	14.019	<0.001	7.298	2.578∼20.658
Abnormal vocalization (vs. normal)	1.647	0.496	11.037	0.001	5.192	1.965∼13.720
Genioglossus: severe thinning (vs. normal)	1.971	0.618	10.163	0.001	7.177	2.137∼24.110
Genioglossus: mild thinning (vs. normal)	0.724	0.572	1.601	0.206	2.062	0.672∼6.327
NRS 2002 ≥ 3 (vs. < 3)	1.332	0.487	7.469	0.006	3.789	1.458∼9.849

### 3.4 Construction of the clinical classification model

Based on the logistic regression results, the prediction model equation was established: Logit(P) = 1.332 × (NRS 2002 ≥ 3) + 1.971 × (Severe Genioglossus muscle thinness) + 1.385 × (presence of comorbidities) + 1.647 × (inability to vocalize) + 1.988 × (voluntary cough ability score ≤2) – 4.551. A nomogram for predicting sarcopenic dysphagia was constructed from five independent risk factors: comorbidities, voluntary cough ability, vocalization, genioglossus muscle thickness, and NRS 2002 score. The probability of dysphagia can be estimated by summing the scores across indicators (Figure 1).

**Figure 1:**
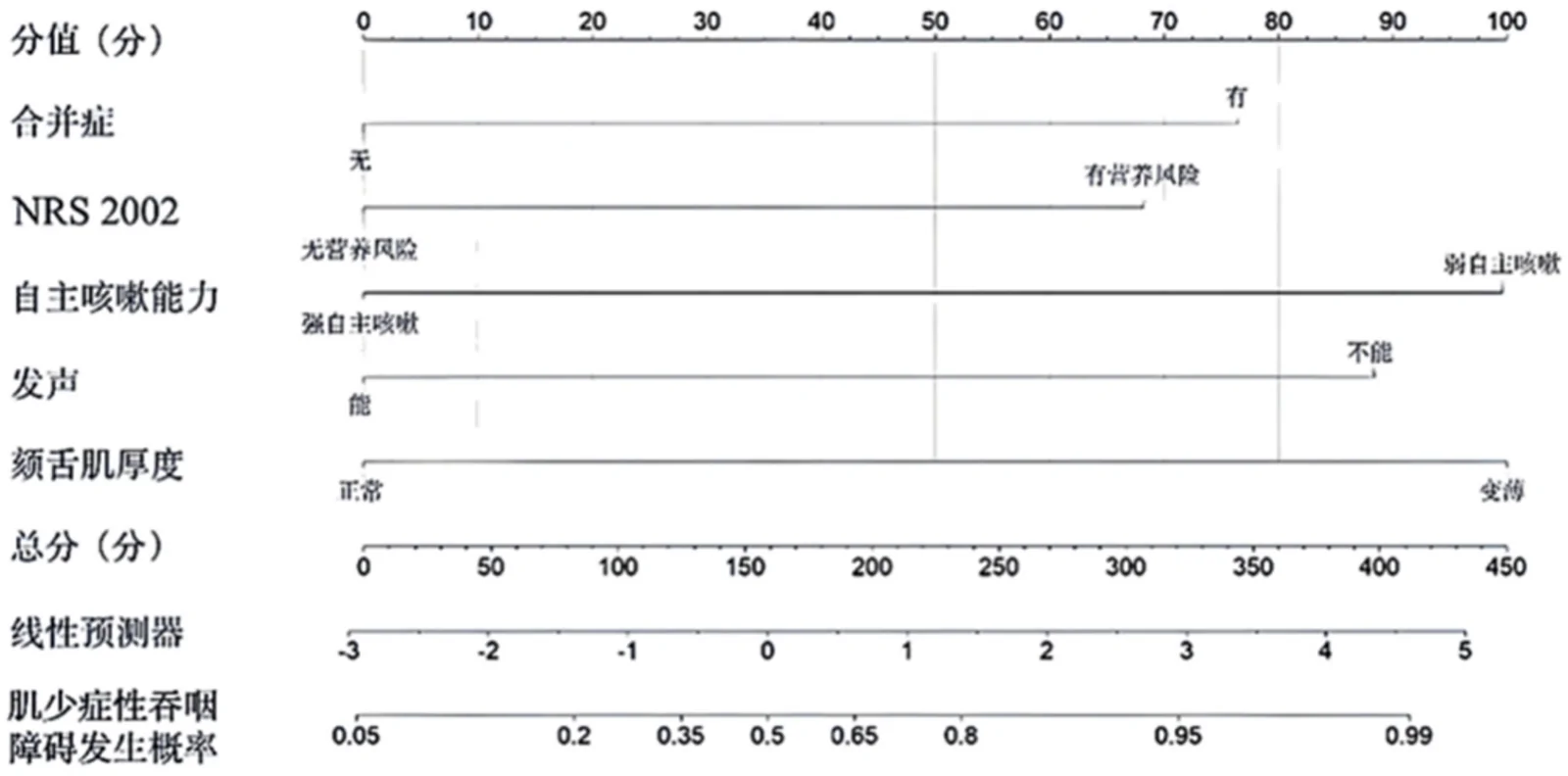
Nomogram for classifying probable sarcopenic dysphagia in older ICU patients.

### 3.5 Validation of the clinical classification model

The calibration plot showed good agreement between predicted and observed probabilities of probable SD, with a mean absolute error of 0.037, a calibration intercept of 0.012, and a calibration slope of 0.94 (Figure 2).

**Figure 2:**
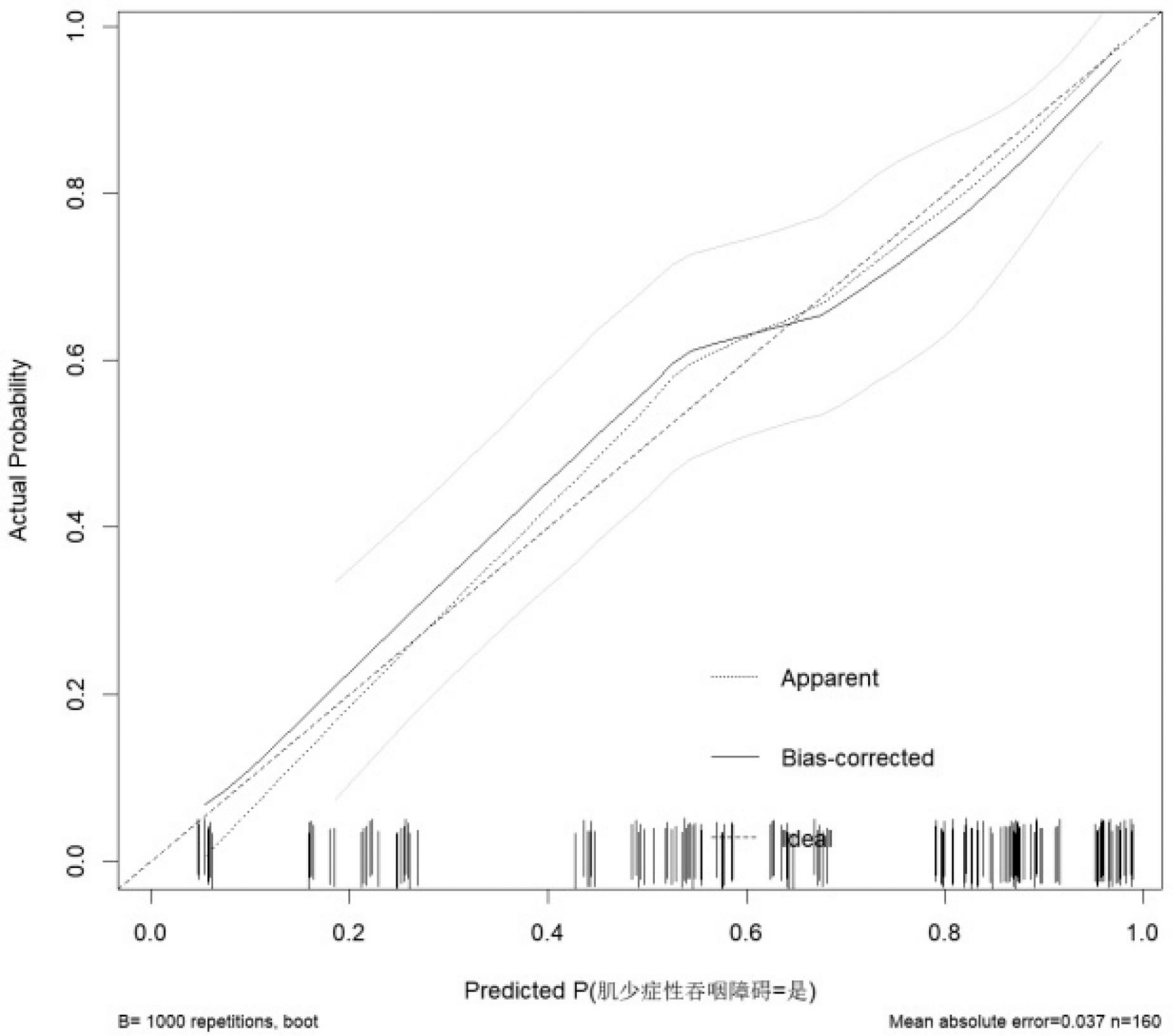
Calibration plot of the prediction model for sarcopenic dysphagia in elderly ICU patients.

Bootstrap internal validation (1,000 repetitions) yielded an apparent AUC of 0.837 (95% CI: 0.775–0.900) and a bias-corrected AUC of 0.812 (Figure 3).

**Figure 3:**
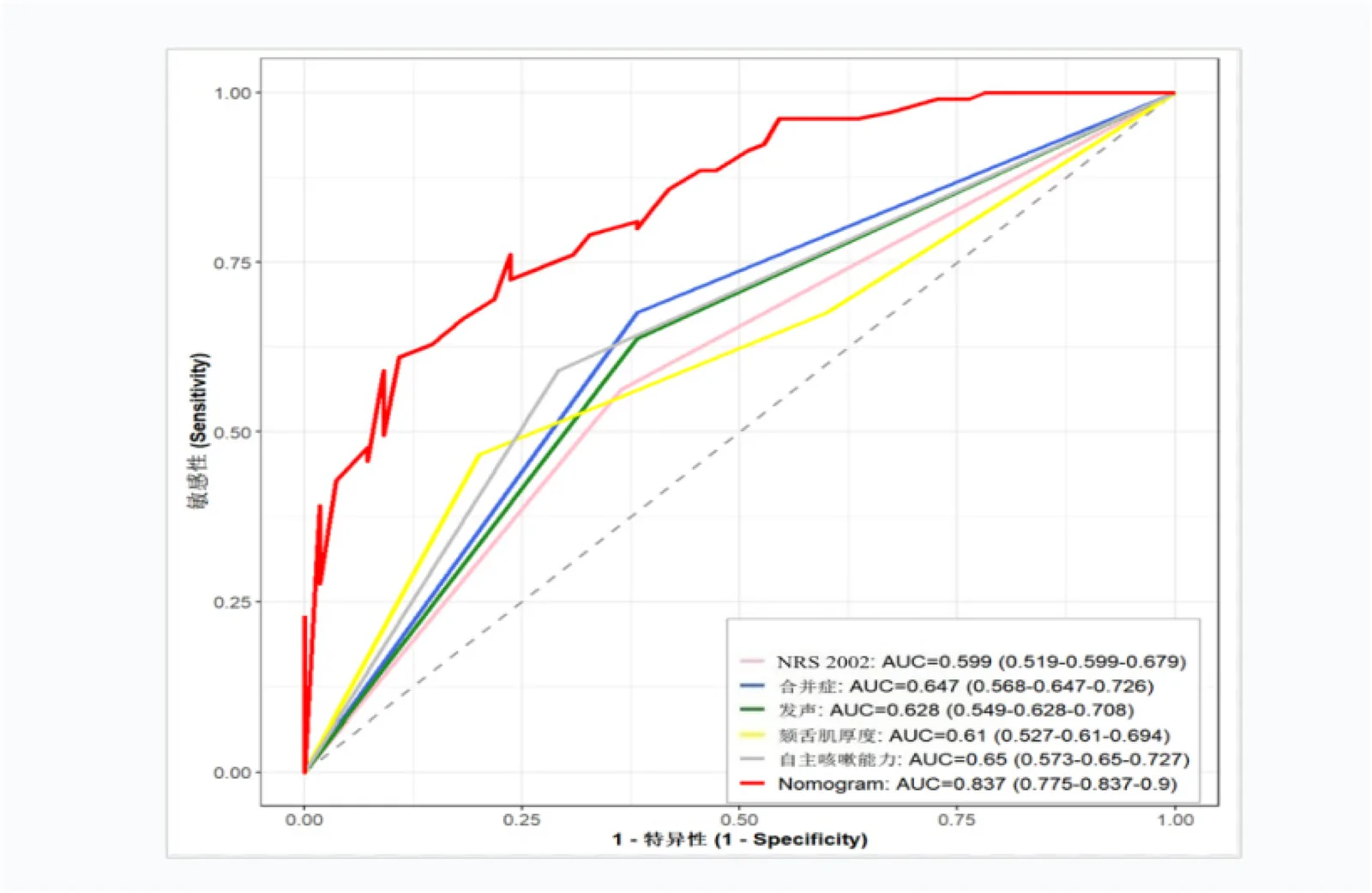
Receiver operating characteristic curve of the prediction model for sarcopenic dysphagia in elderly ICU patients.

Decision curve analysis showed that the nomogram curve lies above both the “treat all” and “treat none” baselines across a wide range of threshold probabilities, indicating positive net clinical benefit (Figure 4). The clinical impact curve demonstrated good high-risk identification efficiency and event capture capability within the 0–0.4 threshold range (Figure 5).

**Figure 4:**
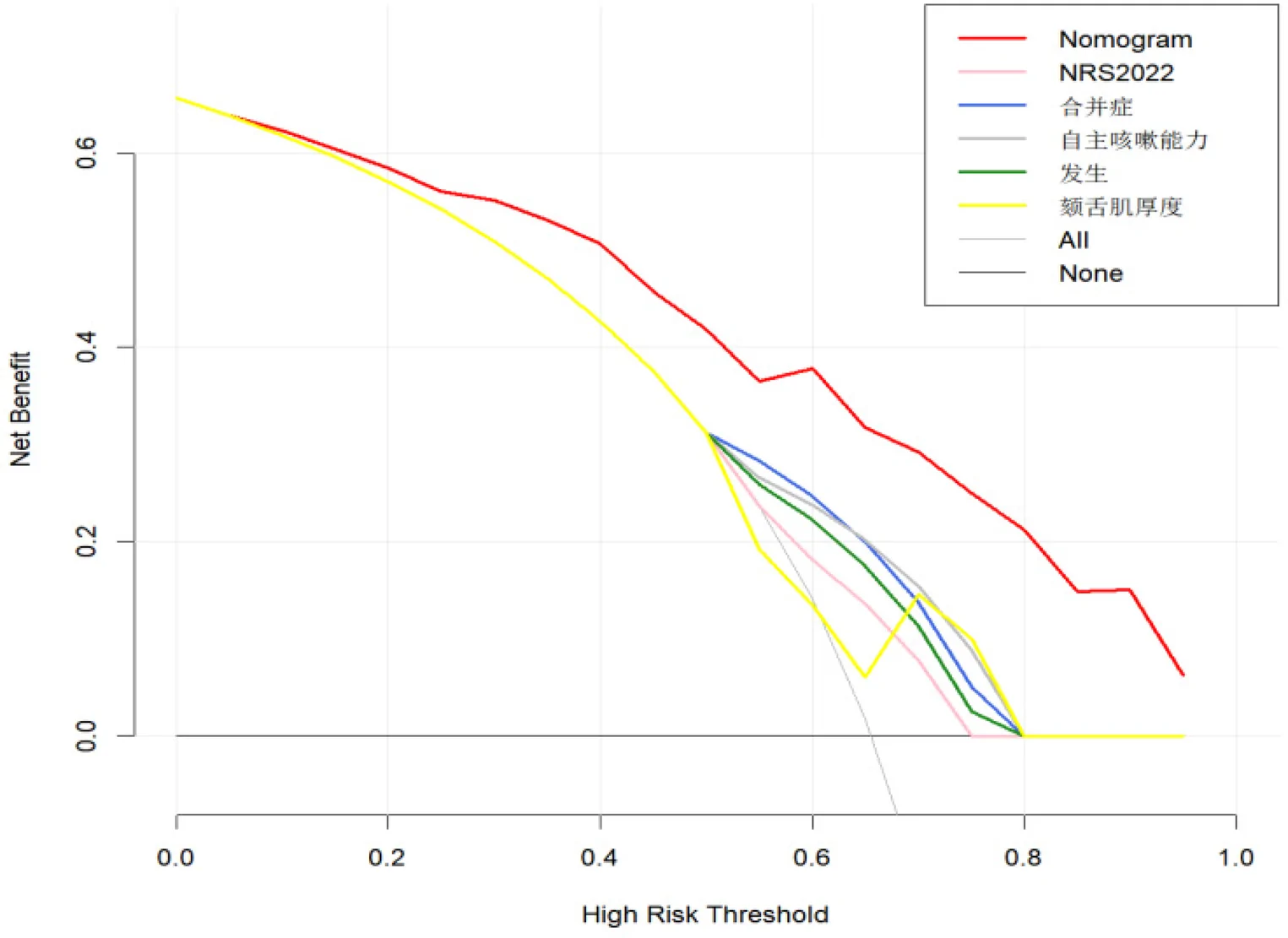
Decision curve analysis of the prediction model for sarcopenic dysphagia in elderly ICU patients.

**Figure 5:**
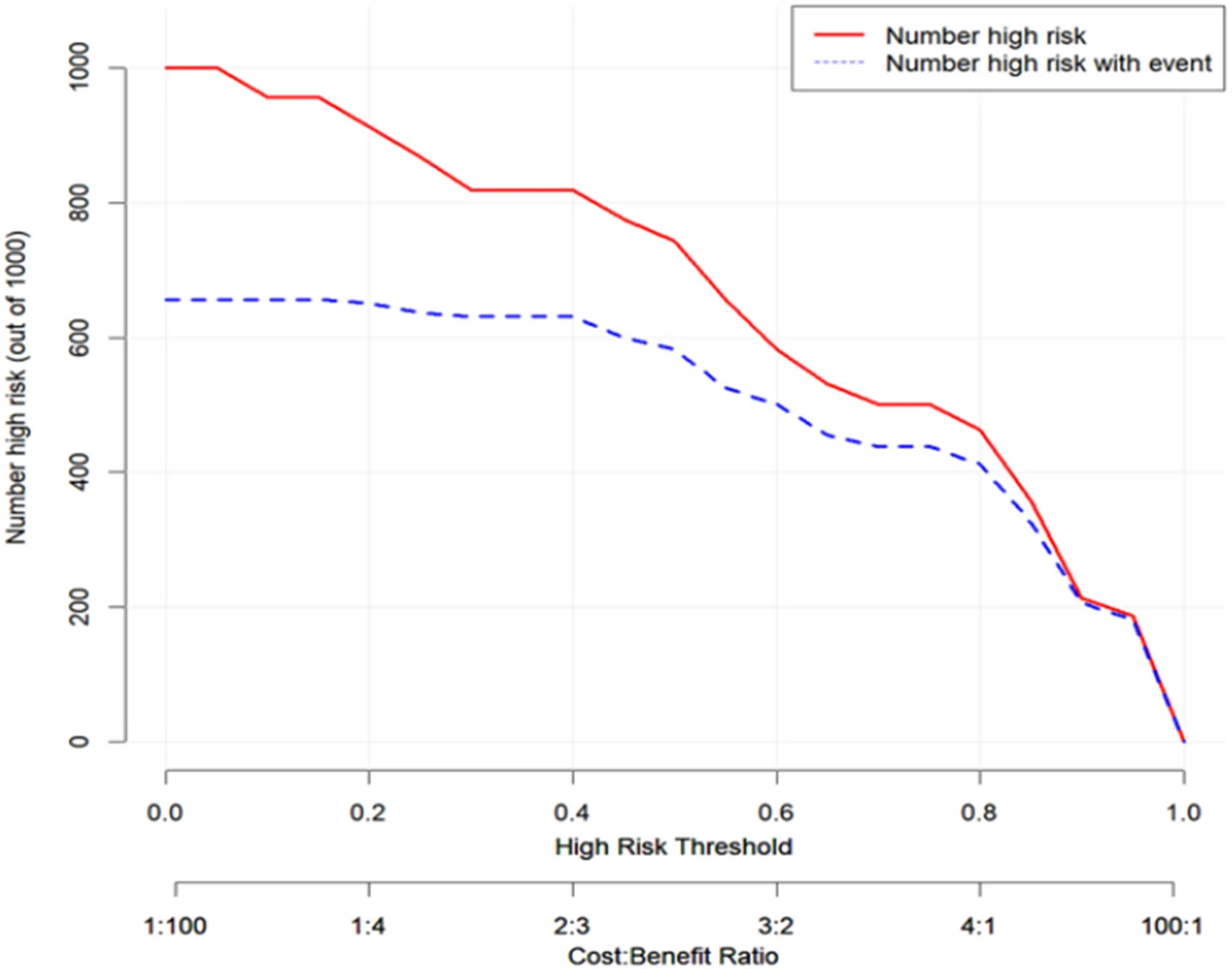
Clinical impact curve of the prediction model for sarcopenic dysphagia in elderly ICU patients.

## 4 Discussion

### 4.1 High prevalence of probable sarcopenic dysphagia in older ICU patients

This cross-sectional study found that the prevalence of probable sarcopenic dysphagia (defined by concurrent positive sarcopenia and dysphagia screening) among older ICU patients was 65.6%, markedly higher than rates reported in general hospitalized older adults (∼30%) and institutionalized older adults (∼45%) (5, 16).

This difference likely reflects the unique ICU environment. Older critically ill patients face dual insults: the direct effects of acute critical illness and secondary ICU-acquired weakness. These factors together drive the co-occurrence and interaction of generalized sarcopenia and localized swallowing muscle dysfunction (6). Key ICU-related contributors include diaphragmatic dysfunction from mechanical ventilation, suppression of swallowing reflexes by sedatives, disuse muscle atrophy from prolonged immobilization, and accelerated protein catabolism under hypermetabolic stress (17). Age-related declines in oropharyngeal nerve, muscle, and salivary gland function further compound vulnerability.

These findings underscore the need for routine swallowing and sarcopenia screening in older ICU patients at admission. Early identification of at-risk patients may help avoid unnecessary fasting and nutritional depletion, and reduce iatrogenic deterioration of swallowing muscle function.

### 4.2 Analysis of associated factors

#### 4.2.1 Nutritional risk is independently associated with probable sarcopenic dysphagia

This study found that patients with NRS 2002 score ≥ 3 had 3.789 times higher odds of meeting the study definition of probable SD than those with lower scores, consistent with previous reports (3). Atrophy of skeletal and swallowing muscles reduces intake of protein, vitamins A and E, and riboflavin, predisposing to malnutrition (18). Insufficient nutrient supply disrupts calcium homeostasis, suppresses IGF-1 production, and fuels chronic low-grade inflammation, which in turn accelerates muscle breakdown and worsens sarcopenia (19). Reduced chewing ability also leads to avoidance of high-nutrient foods such as meat and fruit, perpetuating a cycle of dietary imbalance and muscle loss (20).

#### 4.2.2 Genioglossus muscle thinning is associated with probable sarcopenic dysphagia

Severe genioglossus thinning was strongly associated with higher odds of probable SD. The genioglossus is the primary tongue abductor, and its thickness correlates closely with tongue protrusion force and bolus propulsion efficiency, along with the mylohyoid and geniohyoid muscles (21). ICU patients are prone to disuse atrophy of the genioglossus due to prolonged bed rest, sedation, and mechanical ventilation, leading to reduced muscle endurance and strength (22).

Bedside ultrasound offers a noninvasive, quantitative method to assess swallowing muscle morphology and may be a useful adjunct to clinical swallowing evaluation. Dynamic monitoring of genioglossus thickness may help track disease progression and rehabilitation response.

#### 4.2.3 Reduced airway protective capacity is associated with probable sarcopenic dysphagia

Weak voluntary cough (score ≤ 2) and abnormal vocalization were both independently associated with higher odds of probable SD, with adjusted ORs of 7.298 and 5.192, respectively. Cough and vocal function are key clinical markers of airway protection, reflecting respiratory muscle strength, vocal fold closure, and neural reflex coordination (23).

Impaired cough prevents effective clearance of aspirated material, increasing pneumonia and atelectasis risk and creating a vicious cycle of dysphagia–aspiration–pneumonia–respiratory failure. Vocal dysfunction often indicates incomplete vocal fold closure or insufficient respiratory support, and is an important early warning sign for silent aspiration. Previous work has shown that vocal dysfunction shares a neuromuscular basis with sarcopenic dysphagia and is modulated by similar pathological processes (24).

#### 4.2.4 Comorbidity burden is associated with probable sarcopenic dysphagia

Patients with multiple comorbidities had 3.995 times higher odds of meeting probable SD criteria. This association may be mediated by inflammatory and pharmacologic pathways (25). Chronic conditions such as diabetes, COPD, heart failure, and chronic kidney disease induce a persistent low-grade inflammatory state with elevated TNF-α and IL-6, which promote muscle protein breakdown, inhibit synthesis, and directly impair swallowing muscle function (26). In addition, polypharmacy in patients with multimorbidity—including sedative-hypnotics, opioid analgesics, and muscle relaxants—can suppress central nervous system control of swallowing and worsen dysfunction (27).

### 4.3 Performance and clinical value of the classification model

The five-variable classification model achieved an apparent AUC of 0.837, which exceeds the predictive accuracy of any single indicator (AUC 0.599–0.650), demonstrating that multifactorial assessment improves classification performance. The visualized nomogram provides ICU clinicians with a simple bedside tool to estimate probability of probable SD, stratify risk, and guide targeted evaluation.

It should be emphasized that this is a cross-sectional classification model, not a prognostic prediction model. The model identifies concurrent probable SD during the same ICU admission and cannot be used to infer causal relationships or predict future onset.

### 4.4 Limitations

This study has several limitations that should be acknowledged:

1. Single-center convenience sampling limits external generalizability. Multicenter studies are needed to verify model performance.
2. Screening-based outcome definition: The Ishii score is a sarcopenia screening tool, not a diagnostic gold standard (e.g., AWGS 2019/EWGSOP2 criteria with body composition analysis). The MWST is a bedside swallowing screen with known false-positive and false-negative rates compared to FEES/VFSS and cannot detect silent aspiration. The operational definition reflects concurrent positive screening results, not definitive etiologic diagnosis of sarcopenic dysphagia; outcome misclassification may exist.
3. Measurement uncertainty: Ultrasound measurements are operator-dependent and may be influenced by patient edema, fluid overload, and probe pressure. Inter-rater and intra-rater reliability were not formally evaluated.
4. Exploratory model: Predictor selection was partially data-driven based on univariate screening. Categorization of continuous variables, while clinically convenient, may have reduced information content and affected model stability.
5. Control group heterogeneity: The control group included patients with neither abnormality, isolated sarcopenia screening positivity, and isolated dysphagia screening positivity.
6. Cross-sectional design: Only associations, not causal relationships, can be established.
7. Assessor blinding was implemented for MWST but not for all measurements, which may introduce detection bias.

### 4.5 Clinical implications

Based on existing literature and clinical practice guidelines, the following considerations may inform bedside care, though they were not evaluated in the present study:

1. Routine nutritional screening and individualized nutrition support for older ICU patients, with emphasis on adequate protein and micronutrient intake.
2. Early swallowing rehabilitation for patients with identified muscle thinning, including tongue resistance training, electrical stimulation, and ice stimulation.
3. Enhanced airway management for patients with weak cough or vocal dysfunction, including 30°–45° semi-recumbent positioning, regular turning and chest physiotherapy, and mechanical cough assistance as needed.
4. Careful review of medication regimens in patients with multimorbidity to minimize drugs that suppress swallowing function.

## 5 Conclusion

This cross-sectional study demonstrates that NRS 2002 score ≥ 3, severe genioglossus muscle thinning, presence of comorbidities, abnormal vocalization, and weak voluntary cough ability are independently associated with probable sarcopenic dysphagia in older ICU patients. The five-variable clinical classification model exhibits acceptable discrimination, calibration, and clinical utility in the development sample, and the accompanying nomogram provides a convenient bedside tool for early risk stratification. Given the exploratory nature of the model, the screening-based outcome definition, and the single-center design, further multicenter external validation is required before widespread clinical application.

## Data Availability

The original contributions presented in the study are included in the article/Supplementary Material, further inquiries can be directed to the corresponding authors.
